# An open phase I/IIa study evaluating safety, patient-reported outcomes and voice function after surgery, local administration of mesenchymal stromal cells and voice training in patients with vocal fold scarring and dysphonia

**DOI:** 10.1186/s13287-026-05022-4

**Published:** 2026-04-19

**Authors:** Erika Bergström Börlin, Ulrika Nygren, Maria Södersten, Svante Granqvist, Nadir Kadri, Ida Rasmusson Duprez, Katarina Le Blanc, Stellan Hertegård

**Affiliations:** 1 Division of Speech and Language Pathology, Department of Clinical Science, Intervention and Technology, Karolinska Institutet, Stockholm, Sweden; 2 Speech and Language Pathology, Medical Unit Allied Health Professionals, Karolinska University Hospital, Stockholm, Sweden; 3 Division of Ear, Nose and Throat Diseases, Department of Clinical Science, Intervention and Technology, Karolinska Institutet, Stockholm, Sweden; 4 Ear, Nose and Throat Diseases, Medical Unit, Karolinska University Hospital, Stockholm, Sweden; 5 Department of Laboratory Medicine, Karolinska Institutet, Stockholm, Sweden; 6 Center of Allogeneic Stem Cell Transplantation and Cellular Therapy (CAST), Karolinska University Hospital Huddinge, Stockholm, Sweden; 7 Department of Biomedical Engineering and Health Systems, School of Engineering Sciences in Chemistry, Biotechnology and Health, KTH Royal Institute of Technology, Stockholm, Sweden

**Keywords:** Vocal fold scarring, Dysphonia, Mesenchymal stromal cells, Voice training, Voice disorders, Local injection, Autologous, Bone marrow, Voice Handicap Index, Vocal fold surgery

## Abstract

**Background:**

Damage to the vocal folds can result in scarring, leading to chronic, severe voice impairments for which lasting and effective treatments are currently lacking. The aim of this clinical trial was to evaluate the safety and effectiveness of autologous bone marrow-derived Mesenchymal Stromal Cell (MSC) therapy for patients with vocal fold scarring and severe dysphonia. Additionally, the study sought to propose a post-operative voice training protocol and explore its potential role in facilitating voice improvement.

**Methods:**

Eight patients with vocal fold scarring and chronic dysphonia underwent surgical scar resection and autologous MSC injection, followed by voice training. Safety was continuously monitored for up to 36 months postoperatively. Data to evaluate therapeutic efficacy was collected pre-treatment, 3 and 12 months post-treatment. Assessments included analysis of vocal fold vibrations, Phonation Threshold Pressure, and Maximum Phonation Time. Patient-reported measures were collected using the Voice Handicap Index, the Vocal Fatigue Index, and ratings of major symptoms and their impact on daily life. Treatment effectiveness was analyzed at both group and individual levels, with clinically relevant changes predefined.

**Results:**

No treatment-related side effects were reported within the 36 months of follow-up. Group-level analysis of the self-reported outcomes indicated positive treatment effects, with Voice Handicap Index scores reduced by −25*.*9 points (95% CI [−48, −3*.*6]) between pre-treatment and 12 months post-treatment. Group-level aerodynamic changes were small with Phonation Threshold Pressure showing a marginal positive change on average (−0.94 cmH_₂_O), as did Maximum Phonation Time (+ 0.2 s). At the individual level, clinically relevant improvements were observed in 63–88% of patients depending on the parameter analyzed. Three patients (38%) achieved relevant improvement on ≥ 5/6 selected parameters in combination. All participants in voice training reported reduced vocal strain following training.

**Conclusions:**

This preliminary, uncontrolled study indicates that local administration of autologous bone marrow-derived MSCs appears safe and is associated with clinically relevant patient-reported outcome improvements in a majority of patients. Larger, controlled trials are needed in the future to establish efficacy and possibly disentangle contributions of MSCs versus voice training.

***Trial registration*:**

This clinical trial is registered in ClinicalTrials.gov (NCT04290182)

**Supplementary Information:**

The online version contains supplementary material available at 10.1186/s13287-026-05022-4.

## Background

The vocal folds comprise three main layers: the vocalis muscle, the lamina propria, and the epithelium. The epithelium and superficial lamina propria vibrate independently of the deeper layers and the vocalis muscle. Mechanical stress, environmental factors, and pathological conditions can impair vocal fold pliability, leading to a range of voice disorders [1, 2].

Injury or damage to the vocal folds can result in scarring, causing stiff tissue that restricts lamina propria vibration and pliability, impairs vocal fold closure, and disrupts the vertical glottal mucosal wave essential for voice production [3]. Known causes include surgery, radiation therapy, phonotrauma, congenital or acquired conditions, severe inflammation, and infections. Vocal fold scarring often leads to severe dysphonia, vocal strain, and vocal fatigue [4, 5].

Behavioral voice therapy is typically the first-line treatment, aiming to balance and strengthen voice subsystems while reducing hyperfunctional compensatory behaviors. However, its effectiveness in reversing scarring is limited [4, 5]. Surgical scar resection often yields suboptimal results and may even worsen the condition [5]. Injections of biomaterials like hyaluronic acid and fat have demonstrated temporary improvements [6, 7]. Animal studies suggest that growth factor injections, such as hepatocyte or basic fibroblast growth factor, may improve vocal fold healing [8, 9]. Several clinical studies have shown promising results. For example, Mattei et al. showed significant improvement in Voice Handicap Index 12 months after injecting 8 patients suffering from vocal fold scarring with autologous stromal vascular fraction [10]. Ma et al. reported significantly improved mucosal waves and voice quality in 8 patients 12 months after repeated injections with autologous fibroblasts into the vocal fold [11].

Mesenchymal stromal cells (MSC) exhibit anti-inflammatory and regenerative properties that promote healthy tissue regeneration and reduce scarring [12]. Animal studies have shown that MSC injections improve vocal fold elasticity and healing while reducing inflammation [13–15]. In the first phase I/II clinical study with a one-year follow-up, Hertegård et al. evaluated the safety and efficacy of MSC treatment [16]. Sixteen patients underwent scar resection followed by autologous MSC injection. All patients were recommended post-operative voice training, but compliance, the number of sessions and content were not documented. No serious adverse events were reported and improved vocal fold function was observed in 12 patients, with 8 showing clinically relevant improvement in Voice Handicap Index scores. These results suggest that MSC treatment may be a safe and feasible option for severe vocal fold scarring [16]. Post-operative voice therapy aims to restore vocal fold biomechanical function by optimizing tissue mechanics, preventing hyperfunctional behaviors, and reducing inflammation [17]. Exercises like resonant voice training and semi-occluded vocal tract techniques, such as tube phonation, have been shown to enhance vocal efficiency and reduce tissue collision forces, potentially aiding vocal fold healing [18, 19]. However, no standardized post-operative voice therapy protocol exists, highlighting the need to develop a post-operative evidence-based protocol. In the present study, we further evaluated therapeutic efficacy for MSC treatment in combination with post-operative voice training to restore voice function in patients with severe dysphonia and vocal fold scarring. Moreover, safety follow-up time was prolonged to 36 months after treatment.

## Method

### Aim

This study aimed to evaluate the safety and effectiveness of local autologous MSC injections for patients with vocal fold scarring and severe chronic voice problems. Specifically, the objectives were to determine the proportion of patients with clinically relevant improved voice function (based on instrumental analyses), reduced subjective voice symptoms, and decreased voice-related social limitations in daily life. Additionally, the study sought to propose a post-operative voice training protocol and preliminarily evaluate its effect on relieving vocal strain, as well as patient compliance and experiences with the training.

### Study design

**Fig. 1:**
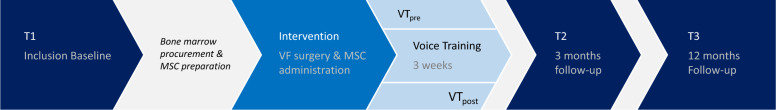
Representation of the study design showing the intervention, voice training and the three timepoints (T) for data collection

This open, single-arm, phase I/IIa clinical trial followed a repeated-measures design (Fig. 1). The study conformed with ICH Guideline for good clinical practice E6(R2) and was approved by the Swedish Ethics Committee (DNR 2019–06160) and the Swedish Medicinal Product Agency (EudraCT:2019-001180-73). The study is registered in ClinicalTrials.gov (NCT04290182).

### Participants

Participants were patients recruited from January 2021 until November 2022. Inclusion criteria were (a) vocal fold scarring; (b) chronic, severe voice symptoms; (c) no active other treatments, and (c) age between 18 and 65 years. Exclusion criteria were (a) ongoing treatment of a laryngeal disorder; (b) active inflammatory condition of the larynx, or papilloma; (c) estimated closure defect > 1.5 mm; (d) disease-free period of   <5 years after malignant disease; (e) smoking; (f) pregnancy or nursing; (g) specific infectious diseases; (h) active or ongoing local or systemic infections; (i) immune suppressive treatment; (j) known hypersensitivity to any of the medicinal product’s excipients.

Eleven patients were assessed for eligibility, two declined to participate, and one withdrew before treatment, leaving a final sample of eight (four women, four men) for per-protocol analysis. Their demographic and clinical details are shown in Table 1. The average age was 44 years. All had experienced severe dysphonia and vocal strain for over a decade, with vocal fold scarring confirmed by microlaryngoscopy. Four patients had unilateral and four had bilateral scarring. The etiologies were sulcus in combination with vocal fold scar (*n* = *7*) and surgery due to laryngeal cancer with radiation therapy (*n* = *1*). Six patients had prior vocal fold surgery, two had received hyaluronan injections more than 10 years ago. The remaining two had no prior vocal fold surgery. All patients had previously received voice therapy.

**Table 1:** Demographic data and clinical information of the patients

	Patients (n = 8)
Age in years, mean (range)	44 (34–63)
Gender (male/female)	4/4
Employed	8
Voice demanding occupation	5
Vocal fold damage*	
Unilateral VF scarring	4
Bilateral VF scarring	4
Medical history	
Asthma	0
Allergies/sinus issues	1
Hearing loss	0
Reflux	0
Prior voice therapy	8
Prior vocal fold surgery	6

^*^7/8 patients had sulcus vocalis in combination with vocal fold scarring

### Procedures

All study procedures were performed at Karolinska University Hospital, Stockholm, Sweden. Autologous bone marrow-derived MSCs were manufactured in accordance with Good Manufacturing Practice (GMP). Autologous bone marrow (30 ± 10 ml) was harvested from each patient and MSCs expanded (see supplementary appendix, pp 1–2 for details). Cells were harvested at passage 1 or 2 and frozen in 10% DMSO. Release criteria included positivity for MSC markers and absence of hematopoietic markers. Quality controls related to safety were outsourced to accredited labs. These tests include sterility, endotoxin, mycoplasma, and genomic stability assessed by karyotyping.

### Vocal fold surgery and local injection of MSCs

Vocal fold surgery was performed under general anesthesia as outpatient care as previously described by Hertegård et al. [16]. During microlaryngoscopy, the degree of vocal fold scarring was assessed, and scar tissue dissected from the lamina propria and either removed or reduced to create a fresh wound. MSC injections (0.5 × 10^6^–1 × 10^6^ cells/vocal fold in 2 × 10⁶/ml infusion solution, consisting of saline for injection supplemented with 10% human serum albumin) were administered into the deep lamina propria and superficial thyroarytenoid muscle using a Medtronic Xomed 27G laryngeal injector. Seven patients underwent unilateral surgery on the most scarred vocal fold, while one patient, where microlaryngoscopy showed symmetrical bilateral scar had bilateral surgery and MSC injections (see Table 3 for details). Postoperatively, patients were advised 5–7 days of relative voice rest. During this period of relative voice rest the patients were advised to limit phonation and only use soft voice, avoid whispering, coughing and throat clearing.

### Monitoring of complications and side effects

Healing assessments, including ear-nose and throat (ENT) status, videolaryngostroboscopy, and side effect monitoring, were conducted one week after surgery. Adverse events (AEs) and Serious Adverse Events (SAEs) i.e. airway swelling, bleeding, or severe infections were tracked at 1 week, and 3, 12, 24, and 36 months postoperatively.

### Voice training

All patients were allocated to participate in post-operative voice training led by a certified Speech and Language Pathologist (SLP). The goal was to optimize conditions for vocal fold healing. Training techniques were based on current evidence suggesting that exercises promote more efficient voice production by reducing hyperfunctional vocal behaviors and attenuating inflammation [18, 20]. The training comprised foundational training [21] and direct facilitation [22] to achieve easy, soft phonation to reduce strain and increase voice flexibility. Exercises focused on Semi-Occluded Vocal Tract Exercises, specifically resonance tube phonation in water [18]. The training lasted three weeks and included 5 individual sessions (duration 30–45 min), initiated 1–2 weeks post-operatively. One participant declined to participate in voice training due to lack of motivation. Patients were instructed to do the tube phonation exercises for 4 min, 5 times per day. The patients filled in a practice sheet on a weekly basis to check their compliance with home practice targets. At the last session, patients were recommended to continue home practice. If the principal investigator (last author) believed that the participant would benefit from prolonged training, they could undertake further voice therapy straight away.

### Data collection and analysis

Data to evaluate treatment efficacy was collected at three time points (T): baseline (T1), three months post-intervention (T2 ± 2 weeks), and 12 months post-intervention (T3; 9–18 months). To evaluate voice training (VT), additional data was collected before (VT_pre_) and immediately after voice training (VT_post_) (Fig. 1).

### Patient-reported measures

Voice symptoms and voice-related impact on daily life were self-reported using the validated voice questionnaires Voice Handicap Index (VHI 30 items; Swedish validation) [23] and Vocal Fatigue Index (VFI; Swedish validation) [24]. In Addition, at T1, patients were asked to write two major voice-related symptoms as well as what consequences those symptoms lead to in daily life i.e., activity limitations and/or reduced quality of life (RoSC, Ratings of individually formulated Major Symptoms and Consequences in everyday life). The patients were then asked to rate the severity of their symptoms and consequences on a 5-point scale with the response options *no problem, slight problem, moderate problem, significant problem, severe problem*. Ratings of the symptoms and consequences were done again at T2 and T3. To evaluate the effect of voice training, the patients reported current status and changes in perceived vocal strain using a questionnaire. The patients’ experiences of voice training were documented using a 10-item survey.

### Evaluation of vocal fold vibrations

General ENT examination, high-speed, and videostroboscopic recordings during phonation were performed at each time point. High-speed recordings were made using a Hispec 1 camera (500 × 250 pixels, 4000 images/s) with a 300 W xenon light source. Videostroboscopy was performed with a Wolf stroboscope and camera, digitized with Picsara systems software and analyzed using High-Speed Studio software. A 70° rigid laryngoscope or videoendoscope was used for examination. The patients sustained the vowel /i:/ at different pitch with a comfortable voice during the examinations. The phonation with the best vocal fold closure, closest to habitual speaking pitch was analyzed. Video recordings were mixed pairwise (T1 and T3) and arranged randomly without sound, with 10% duplicated for intra-reliability testing. Two experienced phoniatricians, blinded to patient diagnoses and treatment, individually judged VF parameters: glottal closure, vibration amplitude, and mucosal wave. The judgements were compared between T1 and T3 using the global categories A (best status), B (worse status), and C (no change/unclear) status for each paired sample. Both judges rated the video samples consistently, demonstrating satisfactory intra- and inter-rater reliability. More than 80% of ratings fell within the same category, with full agreement in 7 of 8 patients.

### Aerodynamic measures

Phonation Threshold Pressure (PTP) was recorded as a measure of perceived vocal effort and as an indirect estimation of glottal mucosal elasticity [25]. PTP was estimated as the intraoral pressure during syllable string production (/pi:/pi:/pi:/) at softest voice possible. Since subglottal pressure (p_sub_) is positively correlated to both Sound Pressure Level (SPL) and fundamental frequency (*f*_o_) [26, 27] the audio signal was captured alongside the pressure recordings as recommended by Patel et al. [28] and visual inspection of pressure peak quality was done to attain valid measures. Three raters did manual measurements of peak height and rated peak quality at two different occasions to allow for intra- and interrater reliability testing (see Fig. 1 in the supplementary appendix for details regarding the measurements). For pressure peak height measurements, the calculated ICC [1, 2] to assess inter-rater reliability was 0.988 [95% CI 0.98, 0.99]. Intra-rater reliability for the three raters ranged between ICC 0.993–0.999. For pressure peak quality ratings, a lower inter-rater reliability ICC of 0.70 [95% CI 0.50, 0.81] was found and intra-rater reliability ranged between ICC 0.81–0.90. PTP for each patient and timepoint was calculated from a mean of 6 individual measurements on 3 adjacent pressure peaks (18 measurements). Measurements of L_eq_ and mean *f*_o_ were made from the phonation between selected pressure peaks. A detailed description of the procedure and materials is available in the supplementary appendix (pp 3–4).

Maximum Phonation Time (MPT) was recorded as a measure of glottal efficiency [29]. Patients sustained an /a:/ vowel after a deep breath, and the longest duration from three trials was used as MPT.

### Statistical analysis and definitions of relevant improvement

Evaluation of the treatment effects was analyzed at group and individual levels. Data was analyzed using R (R core team, 2023). For inferential statistics we focused on Confidence Intervals (CIs). Given the challenges of interpreting treatment effects from group analyses [30, 31], the primary focus was evaluation on individual level, with clinically relevant changes pre-defined. Definitions of relevant change can be made by using different approaches such as measurement theory [32], patient reports [33], expert opinions [34, 35], and from utilizing reference data [36].

For Voice Handicap Index we used the cut-off value from the Swedish validation study that indicates relevant change and the cut-off value that indicates a voice disorder [23]. A reduction of ≥ 13 points between T1 and T3 was interpreted as an improved result and a total VHI score below 20 at T3 was defined as substantial improvement. For Vocal Fatigue Index we defined improvement (though not necessarily to a relevant degree) as reduced scores between T1 and T3. Substantial improvement was defined by adopting the suggested thresholds on subscale one and two to separate individuals with vocal fatigue from healthy controls [24] thus, patients who rated below these thresholds at T3 (but not at T1) were considered substantially improved. For RoSC we were interested in whether the patients rated their symptoms and consequences as being less problematic after treatment. Improvement was defined as positive movements on the rating scale, i.e., patients who rated their symptoms and consequences as less problematic were considered improved. Ratings in the category *no* or *mild problems* at T3 compared to T1 were defined as substantial improvement. For the aerodynamic measures a reduction of ≥ 0,88 cmH_₂_O (PTP) and increase of ≥ 1,01 s (MPT) between T1 and T3 were considered a relevant improvement [37, 38]. For the evaluation of VF vibrations, we used expert judgements by the phoniatricians to define clinically relevant change and, therefore, did not measure relative vibration amplitude or glottal area variations with software as in the previous study [16].

Missing data was minimal. One baseline PTP recording was lost due to technical issues, leading to the exclusion of that patient from PTP analysis. One patient was absent at T2, and two questionnaire items were missing (only at T2). Missing T2 data were imputed using the last observation carried forward method, which, while conservative, carries a potential risk of bias [39].

## Results

A Data and Safety Monitoring Board (DSMB) of three independent laryngologists reviewed all reported side effects up to five months post-treatment. No local or systemic AEs or SAEs related to MSC treatment were identified. However, one patient experienced an SAE 16 months after treatment—a laryngeal infection affecting the cricoid cartilage requiring antibiotics and temporary tracheostomy. This patient had a history of unilateral laryngeal cancer (> 15 years prior) and radiation therapy. CT imaging confirmed radiochondronecrosis, and biopsies showed inflammation without malignancy or dysplasia. By the 21-month follow-up, the inflammation had been resolved. The DSMB concluded that the infection was due to the patient’s preexisting risk of local infection from radiochondronecrosis, not the MSC treatment.

### Voice Handicap Index, Vocal Fatigue Index and ratings of symptoms and consequences

Self-reported outcomes indicate a positive treatment effect on average (Table 2). Voice Handicap Index scores decreased (indicating improvement) from 74.8 at T1 to 55.4 at T2, and further to 48.6 at T3, reflecting a mean reduction of 26 points (one-third) for the group. All subscale scores decreased, with the greatest change in the emotional subscale (MD −10), followed by functional (MD −8.25) and physical (MD −7.75) subscales (Table 2).

**Table 2:** Group level statistics on the different outcome measures and time points

	Time points			MD
	T1	T2	T3	
Voice Handicap Index, total	74.8 (20.5) [57.6, 91.8]	55.4 (20.9) [37.9, 72.9]	48.6 (18.6) [33.0, 64.2]	−25.9 [−48.0, −3.6]
Voice Handicap Index, function	24.8 (5.8) [19.9, 29.6]	19.6 (7.3) [13.5, 25.7]	16.5 (7.5) [10.2, 22.8]	−8.25 [−15.7, −0.7]
Voice Handicap Index, physical	26 (4.4) [22.3, 29.7]	19.9 (6.3) [14.6, 25.2]	18.2 (4.3) [14.7, 21.8]	−7.75 [−13.7, −1.8]
Voice Handicap Index, emotional	24 (11.4) [14.7, 21.8]	15.9 (9.2) [8.1, 23.6]	13.9 (8.5) [6.8, 21]	−10.1 [−20.2, −0.1]
VFI; communication and avoidance	32.1 (7.5) [25.9, 38.4]	21.2 (8.8) [13.9, 28.6]	22.4 (6.3) [17.1, 27.7]	−9.75 [−15.9, −3.6]
VFI; pain and discomfort	8.4 (7.1) [2.5, 14.3]	3.9 (3.8) [0.67, 7.1]	4.5 (3.7) [1.44, 7.6]	−3.87 [−9.2, 1.5]
VFI; improvement after voice rest	5.2 (2.3) [3.3, 7.2]	5.2 (3.7) [2.1, 8.4]	3.4 (3) [0.8, 5.9]	−1.87 [−3.7, −0.01]
Phonation Threshold Pressure (PTP)	7.2 (1.6) [5.6, 8.7]	7.06 (1.4) [5.8, 8.4]	6.2 (1.9) [4.5, 8.0]	−0.94 [−3.1, 1.2]
Maximum Phonation Time (MPT)	13.8 (4.6) [10, 17.7]	15.1 (5.5) [10.5, 19.8]	14 (5.1) [9.7, 18.3]	0.2 [−6.5, 6.8]

Data are shown as mean (standard deviation) and [95% Confidence Interval] or MD = mean difference between time points T1 and T3 [95% Confidence Interval]. Voice Handicap Index (VHI) consists of 30 items rated on a 5-point scale resulting in a possible range from 0 (no impact) to 120 (severe impact). The VHI subscales (function, physical, emotional) consist of 10 items each resulting in a possible range of 0 (little impact) to 40 (more impact). Vocal Fatigue Index (VFI); communication and avoidance consist of 11 items resulting in a possible range of 0 (no voice-fatigue related communication impact) to 44 (more voice-fatigue related communication impact). VFI; pain and discomfort consist of 5 items resulting in a possible range of 0 (no discomfort and pain during phonation) to 20 (more discomfort and pain during phonation). VFI; improvement after voice rest consists of 3 items resulting in a possible range of 12 (no improvement of voice rest) to 0 (more improvement of voice rest). Phonation Threshold Pressure (PTP) was measured in cmH_2_O and Maximum Phonation Time duration in seconds (s). *n* = 8 for all outcome measures but PTP where* n* = 7

**Fig. 2:**
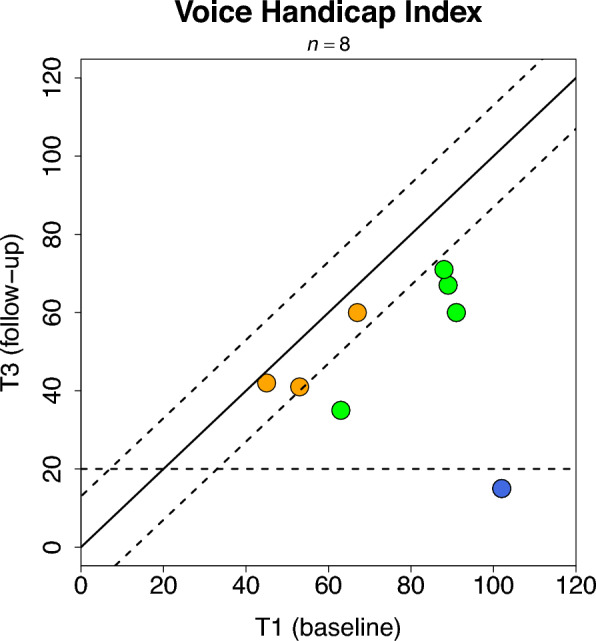
Scatterplot of Voice Handicap Index total scores for each patient at T1 and T3. Each point illustrates one patient´s VHI total score on T1 (x-axis) and T3 (y-axis). The full diagonal line illustrates no difference between T1 and T3. The dotted diagonal lines are thresholds to indicate relevant change (> 13 points from baseline), and the horizontal dotted line is a threshold of 20 points on total VHI score indicating substantial improvement. Green dots are data from patients who improved to a relevant degree, orange dots from those who did not change, and the blue dot is the data from the patient who improved substantially

Voice Handicap Index (VHI) improved to a relevant degree (a decrease of ≥ 13 points) after treatment in 5 of 8 patients (63%), (Fig. 2). A 95% CI was calculated around this proportion [0.24, 0.91] indicating that 24–91% of future patients undergoing this treatment could improve on the VHI scale based on our result. One patient (13%; 95% CI [0, 0.6]) showed substantial improvement (scored below 20 at T3).

**Fig. 3:**
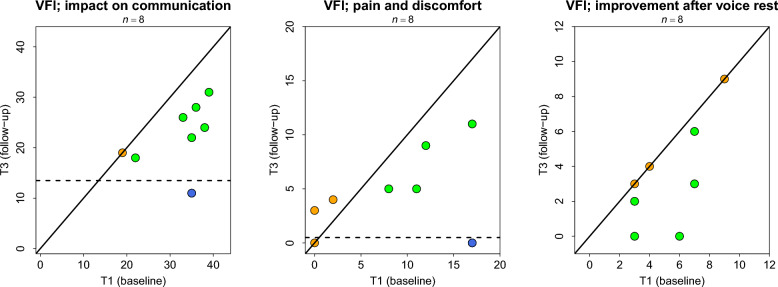
Scatterplots of Vocal Fatigue Index subscale scores for each patient at T1 and T3. Each point in the separate plots illustrates patient’s Vocal Fatigue Index (VFI) subscale score on T1 (x-axis) and T3 (y-axis). The full diagonal line illustrates no difference between T1 and T3. The dotted horizontal lines are thresholds to indicate substantial improvement. The colored dots indicate participants who improved (green, though not necessarily to a relevant degree), remained unchanged (orange) or improved substantially (blue)

Vocal Fatigue Index subscale one (impact of vocal fatigue on communication), improved in 7 out of 8 patients (88%), of which one (13%; 95% CI [0, 0.5]) met the threshold for substantial improvement (scored below 13.5 points at T3). Subscale two (pain/discomfort during phonation) improved in 5 of 8 patients (63%), with one (13%; 95% CI [0, 0.5]) achieving substantial improvement (scoring below 1.5 at T3). Subscale three (improvement after voice rest) improved in 5 of 8 (63%) patients, while 3 remained unchanged (Fig. 3).

At group-level, impact of vocal fatigue on communication (VFI subscale one) decreased by approximately one-third (MD −9.75). Discomfort and pain during phonation (subscale two) showed less reduction (MD −3.87) and was not statistically robust (95% CI [−9.2, 1.45]). Improvement after voice rest (subscale three) showed a positive effect (MD −1.87) (Table 2).

**Fig. 4:**
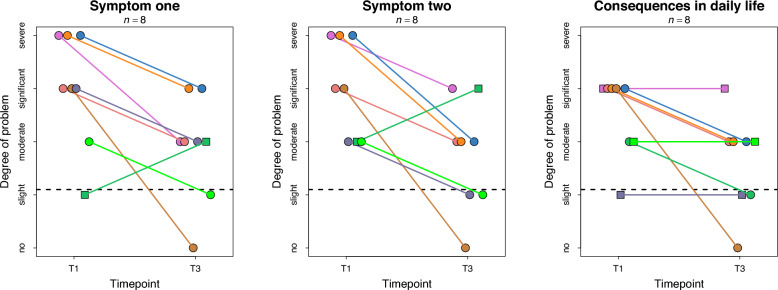
Individual trajectories for Ratings of Symptoms and Consequences (RoSC) between time point T1 and T3. The separate boxes illustrate individual trajectories on the measures from each patient’s formulated symptoms (one and two) as well as consequences in daily life. Each line illustrates one patient’s trajectory between T1 and T3. Ratings of degree of problem (y-axis) were done on a categorical scale with the qualitative different response categories *no*, *slight*, *moderate*, *significant problem,* or *severe problem.* The dotted horizontal lines are thresholds to indicate substantial improvement. Dots show data from patients who improved to a relevant degree whereas squares are from patients with no improvement

Figure 4 shows individual trajectories of the ratings of the major symptoms and consequences in daily life. Ratings of symptom one showed that 7 of 8 patients (88%; 95% CI [0.47, 1.0]) improved to a relevant degree, with 2 of 8 (25%; 95% CI [0.03, 0.65]) achieving substantial improvement. One patient (pG) worsened. Similarly, for symptom two, 7 of 8 patients (88%) improved, and 3 (38%; 95% CI [0.09, 0.76]) improved substantially. One patient (pG) reported worsening. At T1, three patients rated their symptoms as severe, but post-treatment, none reported severe symptoms. At follow-up, 5 of the 8 patients (63%; 95% CI [0.24, 0.91]) showed relevant improvement in daily life consequences, and 2 (25%; 95% CI [0.03, 0.65]) improved substantially. No patients experienced worsening.

### Vocal fold vibrations, phonation threshold pressure and maximum phonation time

Assessment of vocal fold vibrations showed clear improvements in 4 of 8 patients (50%), while 4 showed no change (with judges agreeing on 3). For patient G, one judge observed improved glottal closure, while the other did not, leading to an overall, and conservative, assessment of no change.

Phonation threshold pressure showed relevant improvement (≥ 0.88 cmH_₂_O decrease) in 5 of 7 patients (71%; 95% CI [0.29, 0.96]), while 2 had increased PTP at T3. Among the patients with decreased PTP, fundamental frequency (*f*_o_) increased in 2 (pC, pF) and decreased in 3 (pD, pE, pH). Similarly, L_eq_ increased in the same 2 patients and decreased in the other 3. PTP was on average reduced from 7.2 cmH₂O at T1 to 7.1 cmH₂O at T2, further to 6.2 cmH₂O at T3 (Table 2).

For Maximum Phonation Time, 3 of 8 patients (38%; 95% CI [0.08, 0.75]) showed a relevant improvement after treatment (≥ 1.01 s increase). MPT increased on average from 13.8 s at T1 to 15.2 s after treatment, with a minor decrease of 0.2 s at T3 (95% CI [−6.5, 6.8]) (Table 2).

### Evaluation of treatment effect on different outcome measures in combination

Table 3 presents an overview of individual treatment effects between T1 and T3 for vocal function parameters and patient-reported outcomes in relation to patient demographics, clinical information and treatment. There were positive effects for Voice Handicap Index and Consequences in daily life in 63% (5/8 patients) and improvement for Vocal Fatigue Index subscale one in 88% (7/8 patients) while only a minority (13–38%) improved substantially. The physiological measures showed positive effects for PTP measurements in 71% (5/7 patients), improved vocal fold vibrations in 50% (4/8 patients) and improved MPT measurements in 37% (3/8). When analyzing the outcome measures in combination, our results show that 3 of 8 (38%) patients (pB, pD, pF) made relevant improvements on 5 out of 6 outcome measures and notably, no patient showed a deterioration in their condition when analyzing these selected outcome measures in combination.

**Table 3:** Patient information and results for vocal function parameters and primary patient-reported outcome measures

Patient	Age, sex	Vocal fold damage	Treatment	Side effects	VT	VHI	VFI; communication	Consequences in daily life	VF vibrations	PTP (*n* = 7)	MPT
A	50, male	Sulcus + scar (bilat)	MSC, unilat Total dose 0.8 × 10^6^ cells	None	Yes	89/67*	36/28*	Significant/significant	Improved*	.	10.47/10.79
B	34, female	Sulcus + scar (bilat)	MSC, bilat Total dose 1.0 × 10^6^ cells	None	Yes	91/60*	35/22*	Significant/moderate*	Improved*	6.30/8.84	12.68/13.7*
C	39, female	Sulcus (bilat) + scar right VF	MSC, unilat Total dose 0.8 × 10^6^ cells	None	Yes	63/35*	38/24*	Significant/moderate*	Unchanged^**y**^	5.94/4.59*	15.54/14.1
D	48, female	Sulcus (bilat) + scar left VF	MSC, unilat Total dose 0.8 × 10^6^ cells	None	Partially	102/15**	35/11**	Significant/none**	Unchanged	6.22/5.24*	14.44/18.9*
E	63, male	Scar (unilat left VF)	MSC, unilat Total dose 0.9 × 10^6^ cells	SAE, unrelated	Yes	45/42	19/19	Slight/slight	Unchanged	6.43/5.32*	12.5/12.5
F	47, male	Sulcus (bilat) + scar left VF	MSC, unilat Total dose 0.8 × 10^6^ cells	None	Yes	88/71*	39/31*	Significant/moderate*	Improved*	6.46/5.07*	10.4/9.6
G	50, male	Sulcus + scar (bilat)	MSC, unilat Total dose 0.8 × 10^6^ cells	None	No	53/41	22/18*	Moderate/slight**	Unchanged	8.25/9.06	24.17/8.8
H	39, female	Sulcus (unilat) + scar left VF	MSC, unilat Total dose 0.7 × 10^6^ cells	None	Yes	67/60	33/21*	Moderate/moderate	Improved*	10.47/5.40*	10.35/24.16*

The table shows individual patient (A–H) and treatment information (columns 1–6) and whether a positive treatment effect occurred or not, on different measures (columns 7–12) for each patient. If a measure is marked with *the patient on that row had a positive outcome after treatment. If a measure is marked with **the participant in that row had a substantial treatment effect after treatment. ^**y**^one judge assessed the patient as improved and one judge as unchanged. *VT* voice training, *VHI* Voice Handicap Index, *VFI; communication* Subscale one on Vocal Fatigue Index, *PTP* Phonation threshold Pressure, *MPT* Maximum Phonation Time. Data from PTP for patient A is missing

### Outcomes of voice training

Seven patients were included in the voice training evaluation. One patient attended only three sessions, while the others completed all six. Average home practice compliance was 78% of target, with three patients fully compliant. Before voice training, all patients found voice use either ‘*very’* or ‘*quite strenuous’* and limiting daily life. After voice training, vocal strain decreased for all. Five participants reported voice use as ‘somewhat strenuous’ and one as ‘quite strenuous’. All patients reported that they were satisfied with the voice training and would recommend it to others with similar voice problems.

## Discussion

The safety analysis confirmed that no SAEs or AEs were related to MSC treatment, aligning with previous findings [16] and supporting the safety of MSC therapy for vocal fold scarring.

Patient-reported outcomes showed positive treatment effects, with improved Voice Handicap Index and Vocal Fatigue Index scores, particularly between baseline (T1) and follow-up (T3). All scales, except subscale two on Vocal Fatigue Index (pain and discomfort during phonation), demonstrated statistically robust changes. The smaller effect on subscale two likely reflects the relatively lower baseline ratings, which is not surprising since pain and discomfort are not primary symptoms in patients with vocal fold scarring. Baseline Voice Handicap Index scores (mean: 74) indicate a substantial impact on daily life before treatment. This result is comparable to findings from previous studies reporting high Voice Handicap Index scores ranging between 59 and 80 points in patients with vocal fold scarring [5, 10]. The high Voice Handicap Index at baseline in our study further highlights the severity of the condition and need for effective treatment.

At individual-level, analyses of the patient-reported measures revealed that 63–88% of patients improved after treatment, aligning with results from our previous study in which approximately half of the patients demonstrated Voice Handicap Index improvement following intervention [16].

Notably, Vocal Fatigue Index subscale one (impact of voice fatigue on communication) improved in 7 of 8 patients. However, the change was not necessarily clinically meaningful, as we lack established thresholds for what constitutes a clinically relevant improvement on this scale. The strict definition of substantial improvement—requiring post-treatment scores to match vocally healthy individuals —was only achieved by 1–2 patients depending on the parameter analyzed. Since vocal fold scarring is such a severe condition, the result, that even a few patients rated normal voice function 12 months after treatment, is positive and promising. However, these results also highlight that even improved patients might need further rehabilitation, such as extended voice therapy, after treatment.

Aerodynamic measures showed marginally positive group-level changes, but individual analyses revealed improvements in 5 of 7 patients for PTP and 3 of 8 patients for MPT. These findings highlight the importance of individual-level analysis, especially in small studies to capture nuanced treatment effects. It should be noted that even though the inter- and intra-rater reliability testing for PTP measurements were excellent [40] the average quality of the pressure peaks was low, which may have affected the validity of the measurements. Phonations at the softest possible level with appropriate articulatory settings to produce stable and flat pressure peaks were difficult to achieve for these patients and the PTP results should be interpreted considering this limitation. Vocal fold vibration assessments showed relevant improvement in 4 of 8 patients and, importantly, no one showed a worsening in their condition. However, for patient G, one judge observed improved glottal closure, while the other did not, leading to the assessment of no change. Most patients had bilateral scarring, yet only one received bilateral treatment. More extensive bilateral interventions may have yielded greater improvements. One patient (pH) exhibited relevant improvement in physiological parameters (vocal fold vibration, PTP, MPT) without corresponding self-reported gains. The contradictory results for this patient might possibly be explained by the relatively lower baseline symptom severity or high vocal demands at work.

All participants who participated in voice training reported reduced vocal strain post-training making it plausible, yet unproven, that tube phonation in water contributed to early gains and may aid vocal fold healing. However, the contribution of voice training to overall voice improvement remains unclear, which is a limitation of this study and that warrant further study. The rationale for including post-operative voice training after MSC treatment is the emerging evidence that such training is effective for improving voice [41]. Moreover, it is common practice that patients who undergo vocal fold surgery are referred to receive behavioral voice therapy to optimize conditions for healing post-surgery and reduce vocal strain which was a major complaint of our patients. In the present study, we attempted to evaluate an intervention that patients in the future are most likely to receive, and it would be un-ethical to refrain from referring patients for post-surgery rehabilitation if that is best practice. In addition, we hypothesize that MSC treatment and voice training may work synergistically in the rehabilitation of patients with chronic vocal fold scarring due to restoration of vocal fold function (via MSCs) coupled with behavioral adaptation and optimization (via VT). It is unlikely that voice training alone can restore the biomechanical properties of scarred vocal folds [4]. In fact, all patients in our study had received voice therapy previously without improvement. On the other hand, while MSC treatment holds promise for improving the structural and functional properties, it does not inherently affect how patients use their voice post-treatment. In individuals with chronic dysphonia, suboptimal functional vocal behaviors may be deeply manifest. Even if MSC treatment restores tissue pliability and vocal fold vibration, patients may not automatically adapt their voice behavior to these new conditions. In our study, voice training might have served a dual purpose by possibly supporting the healing process [19] and by targeting functional voice use—by addressing posture, breath support and laryngeal muscle strength and flexibility. This hypothesis is, however, speculative and warranting further investigation. In the previous study [16], the patients were offered voice therapy but since this was not controlled, a direct comparison of the results should be made with this methodological difference in mind.

In summary, based on our results we cannot be sure of the contribution of voice training to overall voice improvement. However, we deem it unlikely that voice training alone contributed to the effect, since all patients had undergone such training before without improvement. We suggest that overall treatment effectiveness should be viewed as a combination of MSC treatment and voice training and speculate that there might be a potential synergistic effect that warrant further investigation for example by using designs with multi factorial arms to isolate effects.

### Methodological considerations

This study’s strengths include the multimodal assessment test battery with detailed safety assessments, use of validated self-report measures, high-speed imaging of vocal fold function, and aerodynamic evaluations. However, as a non-randomized, non-controlled trial, causality cannot be established. The case that we did not include a control group with patients treated with surgery only (without MSCs) also limits the interpretation of the MSC treatment effect. Such a control group would ideally be included in future studies. However, previous animal studies by us and others with such a control group showed a clear positive effect of MSC as compared to surgery/scarring only [4, 13–16].

Although the small sample size severely limits generalizability, and the wide CIs show that our study has a low precision, this study still offers valuable insights by presenting both group-level data and especially detailed individual analyses, highlighting the proportion of patients who achieved clinically relevant improvements. As stated previously, operationalizing definitions for clinically relevant change can be done using different approaches. In the present study we used suggested cut-offs or thresholds provided from previous validation studies [23, 24, 37, 38] as well as expert opinions. For VFI and RoSC measures we had no previous data to utilize when defining relevant change, instead we decided to express the treatment effect as positive movements on scale, a methodological choice that can be seen as arbitrary and should be taken into consideration when interpreting our results. There is apparent need for future research to provide reference data for standard measures used for voice assessment.

This study reinforces the findings of our previous research [16], adding to confirming the absence of treatment-related side effects or complications, along with an indication of positive self-reported voice outcomes for a majority of patients and favorable physiological data even though the PTP results should be interpreted cautiously due to challenges in obtaining high-quality soft phonation peaks in this population. Notably, no patient experienced vocal deterioration. A more detailed analysis of self-reported measures and evaluations in the present study revealed relevant improvements, particularly in parameters related to vocal strain and fatigue—prevalent symptoms among patients with vocal fold scarring.

## Conclusions

The results in this preliminary, uncontrolled study indicate that MSC injection in combination with voice training to treat vocal fold scarring appears safe and is associated with clinically relevant patient-reported improvements in a majority of patients. Despite the study’s methodological limitations, it may represent an incremental advance in this difficult-to-treat condition. Future larger, controlled, studies should establish efficacy of MSC therapy and potentially disentangle contributions of MSCs versus voice training to make further steps towards developing a feasable and efficient treatment option for patients with severe voice disorders to ultimately improve their quality of life.

## Supplementary Information

Supplementary material 1. A supplementary appendix containing supplementary text on bone marrow collection and manufacturing process (supplementary table 1), Phonation Threshold recordings and analyses as well as references.

## Data Availability

The data sets generated and analyzed during this study are not publicly available due to ethical restrictions but may be available upon reasonable request to the corresponding author.

## Abbreviations

AEs: Adverse events
CI: Confidence interval
CT: Computed tomography
DSMB: Data and Safety Monitoring Board
ENT: Ear-nose and throat
*f*_o_: Fundamental frequency
ICC: Intraclass correlation coefficient
L_eq_: Equivalent sound pressure level
MPT: Maximum phonation time
MSC: Mesenchymal stromal cell
P_sub_: Sub glottal pressure
PTP: Phonation threshold pressure
RoSC: Ratings of Symptoms and Consequences
SAEs: Serious adverse events
SLP: Speech and Language Pathologist
SPL: Sound pressure level
T: Time point
VFI: Voice Fatigue Index
VHI: Voice Handicap Index
VT: Voice training
