# Supplementary material for: An open phase I/IIa study evaluating safety, patient-reported outcomes and voice function after surgery, local administration of mesenchymal stromal cells and voice training in patients with vocal fold scarring and dysphonia
Source: Stem Cell Res Ther. 2026 Apr 19;17:146. doi: 10.1186/s13287-026-05022-4 (PMC13094235; doi:10.1186/s13287-026-05022-4)
Supplement: Supplementary file 1 — Supplementary material 1. A supplementary appendix containing supplementary text on bone marrow collection and manufacturing process (supplementary table 1), Phonation Threshold recordings and analyses as well as references. [file 13287_2026_5022_MOESM1_ESM.docx]

**Stem Cell Research and Therapy**

**Supplementary appendix**

**Supplement to:** An Open Phase I/IIa Study Evaluating Safety, Patient-Reported Outcomes and Voice function after Surgery, Local Administration of Mesenchymal Stromal Cells and Voice Training in Patients with Vocal Fold Scarring and Dysphonia

**Table of content**

Page 1: Supplementary material: Bone marrow collection and manufacturing process

Page 2: Supplementary table 1: Quality controls and release criteria of MSC-product KI-MSC-PL-204.

Page 3-4: Supplementary material: Phonation Threshold recordings and analyses

Page 5: Supplementary references

**Bone marrow collection and manufacturing process**

Bone marrow collection and start of production

Bone marrow (BM, 30 ± 10 ml) from each patient was harvested from the posterior iliac crest under local anesthesia in semi-sterile conditions. The BM was placed in sterile tubes containing Dulbecco’s Modified Eagle Medium (DMEM) low glucose supplemented with heparin and transported to the GMP-facility within one hour. Manufacturing of the MSC products was GMP-compliant and performed in class A with a B-class environment.

Mononuclear cells (MNCs) were isolated using a Ficoll isolation procedure. Briefly, BM was washed in phosphate-buffered saline (PBS) and then layered on top of Ficoll solution. After centrifugation, the MNCs phase was collected and suspended in complete medium consisting of 95% DMEM- medium (Life Technologies, United Kingdom) supplemented with 5% γ-irradiated human platelet lysate (PL, PL Bioscience, Germany).

The cell suspension was seeded in sterile cell culture flasks (NUNC easy flasks, Thermo Fisher Scientific) at a density of 160,000 cells/cm² and then incubated at 37°C, 5% CO_2_ and 90 % relative humidity. Non-adherent cells were removed by medium change after 24-72 hours. MSCs were then expanded for 10-16 days with medium change every 3 to 4 days.

MSC expansion and generation of MSC cell product (KI-MSC-PL-204)

When dense clusters have formed, reaching a confluency of 70%, the MSCs were washed with PBS and then detached using TrypLE select (Life Technologies) at 37°C for approximately 5 minutes. MSCs were counted and then reseeded at a density of 3,500–4,000 cells/cm^2^ and further expanded for 7-9 days with 1-2 media changes. When the cells had reached 70% confluent, the MSCs were detached again using TrypLE select. After detachment, the cells were resuspended in complete medium and counted. If sufficient cells were generated (product cells + cells for quality controls) the MSCs were processed into final drug product in passage 1 (DP-P1). If less MSCs were obtained, one additional expansion cycle was performed generating a drug product in P2 (DP-P2). The cell products were cryopreserved in a concentration of 5 x 10^6^ cells/ml in complete medium containing 10% Dimethyl sulfoxide (DMSO, WAK chemie). Cryo vials were placed in Cool cell containers and immediately placed in -80 °C. The vials were moved to a -150°C freezer within 72 hours for long-term storage.

**Quality controls of the MSC-products**

Identity of the MSC-product was based on cell morphology in culture, rate of expansion by calculation of population doubling levels (PDL) and expression of surface markers using flow cytometry. Cryopreserved QC vials were thawed and washed with PBS containing 0,1% Bovine serum albumin (BSA). MSCs (0,1-0,2x10^6^/vial) were incubated for 30 min at +4°C protected from light using the following MAb: CD45-APC, human (clone HI30, γ1, from Biolegend), CD31-PE (Clone L133.1, γ1, from BD), CD14-FITC (Clone M5E2, γ2a from BD), CD34-PerCP/Cy5.5 (Clone 8G12, γ1 from BD) HLA-DR BV421(DR,DP,DQ), Clone Tu39, γ2a, from BD), CD105-FITC (Clone SN6, γ1, from Ancell), CD73-PE (Clone AD2, γ1, from BD) and CD90-PerCP/Cy5.5 (Clone 5E10, γ1, from Biolegend). Live/dead cells were quantified using the Live/Dead Fixable Aqua Dead Cell Dye stain kit (Life Technologies). After staining the cells are washed and fixed in CellFix (BD). Data were acquired on a BD FACSVerse Flow cytometer, by collecting a minimum of 30,000 events and analyzed with the FlowJo software.

Quality controls related to safety were outsourced to accredited labs. These tests include sterility, endotoxin, mycoplasma, and genomic stability assessed by karyotyping.

**Supplementary Table 1. Quality controls and release criteria of MSC-product KI-MSC-PL-204.**

| **Test** | **Method** | **Release criterium** |
| --- | --- | --- |
|  | **Drug Substance** |  |
| Morphology | Microscopy (visual inspection) | Adherent cells, homogenous fibroblastic cell morphology |
| Sterility | European Pharmacopoeia 2.6.1 | Sterile |
| Mycoplasma | European Pharmacopoeia 2.6.7 | Not detectable (< 10 cfu/ml) |
| Endotoxin | European Pharmacopoeia 2.6.14 | Test material: <0.5 EU/ml^1^ |
| Population doubling level (PDL)^2^ | Manual counting | P1 <5; P2 <4 |
| **Drug Product** | | |
| Viability (cryopreserved and thawed sample) | Manual counting | ≥70% |
| Sterility | European Pharmacopoeia 2.6.1 | Sterile |
| Identity | Flow cytometry | CD105 ≥ 50%/ of all live cells  CD73 ≥ 70%/ of all live cells  CD90 ≥ 70%/ of all live cells |
| Purity | Flow cytometry | CD14<5% of all live cells  CD34<5% of all live cells  CD45<5% of all live cells  CD31<5% of all live cells |
|  |  | CD14 + CD34 + CD45 + CD31 < 15% |
| Genomic stability | Karyotyping | 46, XX or 46, XY in 25 metaphases where ≤ 2 nonidentical aberrations are accepted |

^1^This limit is an alert limit and may be exceeded if compliance with the patient-specific limit <5 EU/kg is verified; ^2^PDL was calculated according to the formula PDL = Ln(cells collected/cells seeded)/Ln(2).

**Recording procedure and analyses of Phonation Threshold Pressure (PTP)**

Recordings of Phonation Threshold Pressure (PTP) occurred in a sound treated booth at the Division of Speech and Language Pathology, Karolinska Institutet. In our study, PTP was estimated by measuring the intraoral pressure during stop consonant production. Recordings of the audio signal were captured alongside pressure recordings to control variations in sound pressure level (SPL) and fundamental frequency (*f*_o_) as recommended by Patel et al (1). Two instructors, a phoniatrician (last author) with extensive experience of conducting subglottal pressure measurement and analyses and a SLP (first author) conducted the recordings together. One gave instructions to the patients, and the other handled the recording program and performed real-time visual inspection of the pressure peaks as well as visual inspection of the phonetogram display (dB SPL on the y-axis and *f*_o_ on the x-axis). The visual inspection enabled assessment of pressure peak quality (shape and flatness) and was done to ensure that soft phonation occurred during the vocal task. These actions were taken to attain valid measures.

The patients were seated in the recording room with a head-mounted omnidirectional microphone (DPA, 4066) placed 15 cm away from, and slightly to the side, of their mouths. Pressure (P_s_) was recorded using a 4 mm diameter plastic tube placed in the corner of the patients’ mouths. Pressure recordings were transmitted through and modulated on an 8 kHz carrier signal using a pressure transducer (Glottal enterprises, MS 110), and captured, as well as the audio signal using a Focusrite Scarlett 2i2 audio interface. Initially, patients were instructed to produce stable syllable strings (7 repetitions of /pi/), using habitual pitch and loudness, with legato articulation and adequate lip closure as recommended by Plexico et al (2). This was done to ensure production of valid pressure peaks. Patients were then asked to repeat the task and decrease voice intensity until the phonation ceased. Recording started when patients performed the vocal task at phonation threshold level (softest voice possible) and lasted until at least one stable string of syllables, with flat enough peaks, was produced.

All recordings were done using the software program RecVox ([www.tolvan.com](file:///C:/Users/Erika%20B%C3%B6rlin/OneDrive%20-%20Karolinska%20Institutet/Doktorandprojekt/Stamcellsprojektet/Manus/www.tolvan.com)) with a sampling rate of 44 100Hz. Calibration of SPL was performed before each participant recording. A stable pressure signal of 10cm/H2O and 0 cmH_2_O was produced (Glottal Enterprises, PC-1H) and captured for the pre-analysis demodulation and calibration procedure. Analysis of pressure and acoustic measurements were done in the software program Sopran ([www.tolvan.com](file:///C:/Users/Erika%20B%C3%B6rlin/OneDrive%20-%20Karolinska%20Institutet/Doktorandprojekt/Stamcellsprojektet/Manus/www.tolvan.com)). Prior to analysis, the modulated pressure signal was demodulated using a script. The recordings were, thereafter, calibrated by using the recorded 10 cmH_2_O and 0 cmH_2_O pressure signals (described above).

Data extraction and analysis took place in three stages. In stage one, the selection of syllable strings was done in consensus by the first and last author. Selection criteria were softest possible phonation (assessed by listening to the audio recording) and flat and stable pressure peaks (assessed by visual inspection). The selected pressure peaks were renamed by using a script in Matlab and saved as 8 separate files, all with different randomizations. The files contained three channels to enable measurement of equivalent sound pressure level (L_eq_) in dB, fundamental frequency (*f*_o_) in Hz, and pressure in cmH_2_O (see figure 1). In stage two, measurements of pressure and acoustic variables were done by three researchers. Rater A was an SLP (first author). Rater B, a civil engineer with extensive experience in performing pressure measurement and who had been involved in the technical setup (author four), and rater C (last author), an experienced phoniatrician. All three assessed all selected peaks at two different occasions enabling testing of both inter- and intra-rater reliability. The raters were blinded i.e., they didn't know which participant or what time point the peaks belonged to. Measurements were made according to a protocol to improve reliability. Instructions included how to prepare the peaks for visual inspection (enlargement and stretching) and how to conduct manual measurement. In addition to measuring subglottal pressure (peak height) a quality rating of each pressure peak was done on a 4-point scale where 0 = unusable peak, 1 = poor peak, 2 = good peak, 3 = very good peak. Each assessment occasion (197 peaks) took approximately two hours and was repeated after 1 week.

In stage 3, individual peaks were numbered and peak groups referring to separate syllable strings labelled. The calculated PTP for each patient and test occasion were based on measurements of the peak group with the highest average peak quality.

**Figure 1.** Image illustrating how measurements of subglottal pressure, fundamental frequency and sound pressure level were conducted in Sopran.

**
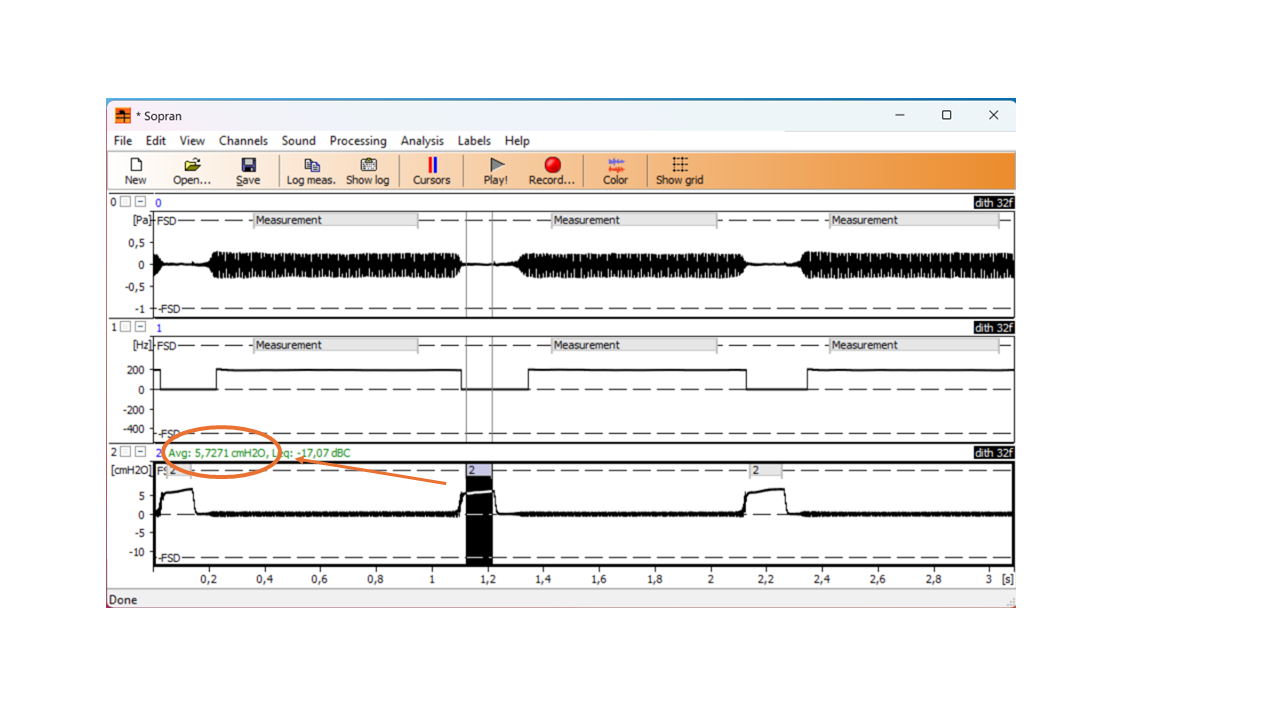

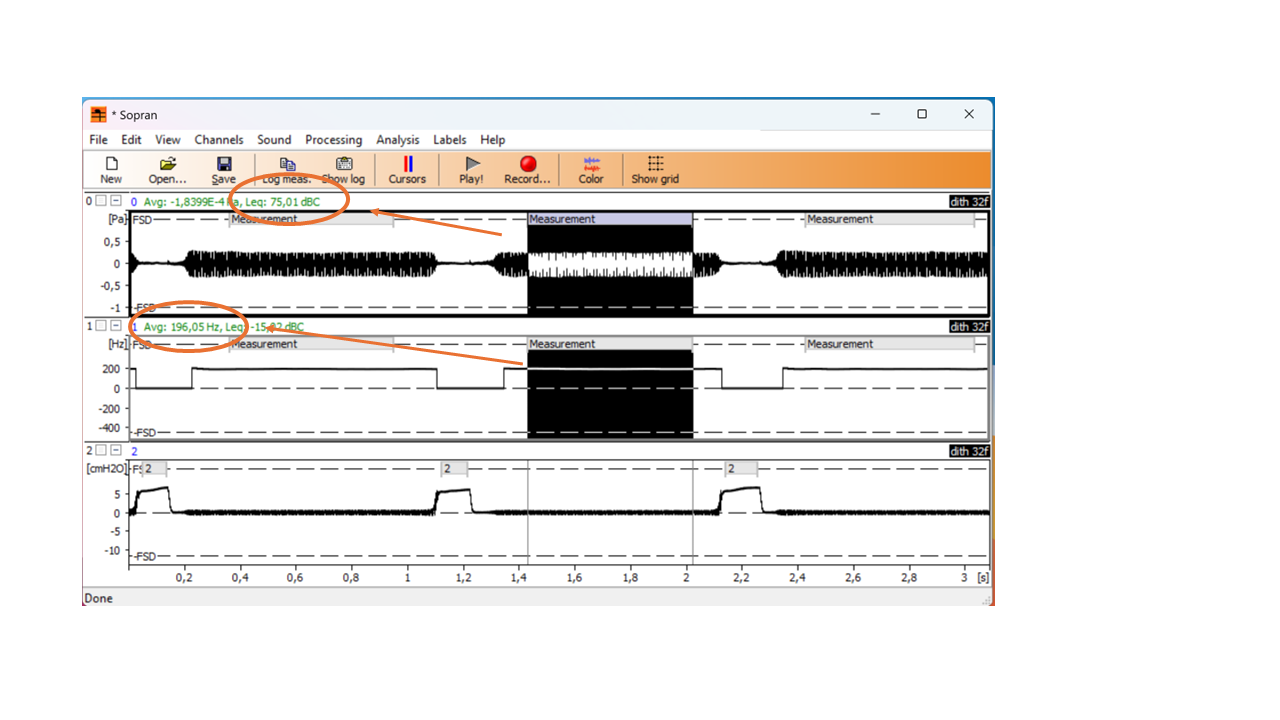
**

The top panel illustrates how manual measurements of average pressure peak height (cmH_2_O) were conducted (bottom channel). The number above the pressure peak is the rated pressure peak quality. The bottom pane illustrates how Sound Pressure Level (L_eq_, top channel) and fundamental frequency (Hz, middle channel) were measured in the vowel following the p-occlusion.

**References (supplementary)**

1. Patel R.R, Awan S.N, Barkmeier-Kraemer J, et al. Recommended Protocols for Instrumental Assessment of Voice: American Speech-Language-Hearing Association Expert Panel to Develop a Protocol for Instrumental Assessment of Vocal Function. *Am J Speech Lang Pathol* 2018; **27**: 887–905.

2. Plexico L.W, Sandage MJ, Faver KY. Assessment of phonation threshold pressure: a critical review and clinical implications. *Am J Speech Lang Pathol* 2011; **20**: 348–66.
